# Supplementary material for: The Impact of Tissue Preparation on Salivary Gland Tumors Investigated by Fourier-Transform Infrared Microspectroscopy
Source: J Clin Med. 2023 Jan 10;12(2):569. doi: 10.3390/jcm12020569 (PMC9864841; doi:10.3390/jcm12020569)
Supplement: Supplementary file 1 [file jcm-12-00569-s001.zip › jcm-2068428-supplementary.pdf]

The impact of tissue preparation on salivary gland tumors investigated by Fourier-transform infrared microspectroscopy

Mona Stefanakis[1,2] and Miriam C. Bassler[1,2*], Tobias R. Walczuch[1], Elena Gerhard-Hartmann[3], Almoatazbellah Youssef[3], Rudolf Hagen[4], Agmal Scherzad[4], Manuel Bernd Stöth[4], Edwin Ostertag[1], Rudolf Hagen[4], Maria R. Steinke[5], Stephan Hackenberg[6], Marc Brecht[1,2**] and Till Jasper Meyer[4**]*

[1]*Process Analysis and Technology (PA&T) Reutlingen University, Alteburgstr. 150, 72762 Reutlingen, Germany*

[2]*Institute of Physical and Theoretical Chemistry University of Tübingen, Auf der Morgenstelle 18, 72076 Tübingen, Germany*

[3]*University of Würzburg, Institute of Pathology, Josef-Schneider-Str. 2, 97080 Würzburg, Germany*

[4]*University Hospital Würzburg, Department of Oto-Rhino-Laryngology, Plastic, Aesthetic & Reconstructive Head and Neck Surgery, Josef-Schneider-Str. 11, 97080 Würzburg, Germany*

[5]*University Hospital Würzburg, Chair of Tissue Engineering and Regenerative Medicine, Röntgenring 11, 97070 Würzburg, Germany*

[6]*Fraunhofer Institute for Silicate Research ISC, Röntgenring 11, 97070 Würzburg, German*

[7]*RWTH Aachen University Hospital, Department of Otorhinolaryngology – Head and Neck Surgery, Pauwelsstr. 30, 52074 Aachen, Germany*

---

[*]These authors contributed equally to this work.

** Corresponding author: Dr. med. Till Jasper Meyer, University Hospital Würzburg, Department of Oto-Rhino-Laryngology, Plastic, Aesthetic & Reconstructive Head and Neck Surgery, Josef-Schneider-Straße 11, 97080 Würzburg, Germany, E-mail: meyer_t2@ukw.de, Phone: +49 931 201 21323, Prof. Dr. Marc Brecht, Hochschule Reutlingen, Alteburgstraße 150, 72762 Reutlingen, Germany, E-Mail: marc.brecht@reutlingen-university.de, Phone: +49 7121 271 2065/2032

Supplementary information

For the evaluation of Fourier-transform infrared band intensity changes and differences in signal relations, FTIR mean spectra of the “native”, “formalin”, “in paraffin” and “dewaxed” tissue were initially baseline-corrected in order to achieve a good comparability. This comparison was performed individually for each parotid tissue type (salivary gland tissue, Warthin tumor, pleomorphic adenoma). An overview of the FTIR mean spectra for each sample preparation and tissue type is illustrated in Figure S1.

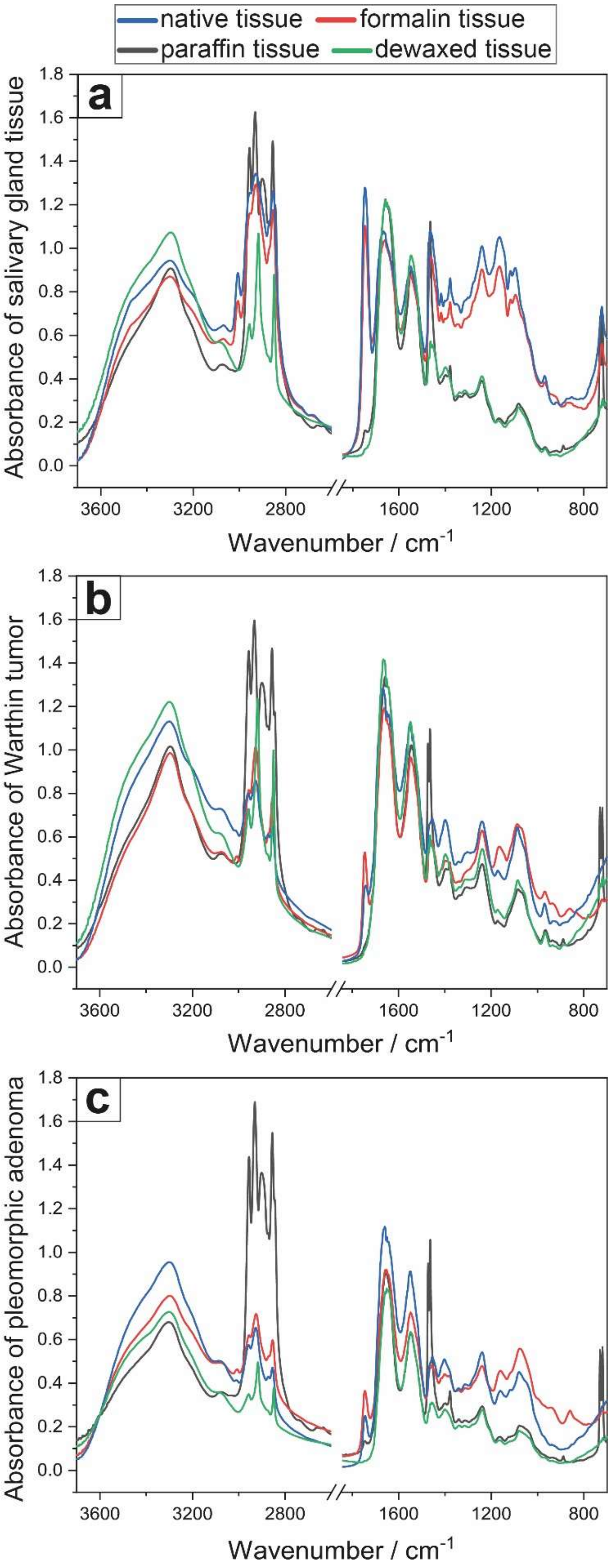

Figure S1. IR band relation and intensity comparison between the different sample preparation methods, illustrated individually for each tissue type: a) salivary gland tissue, b) Warthin tumor, c) Pleomorphic adenoma. All FTIR mean spectra were baseline corrected. The "native tissue", the "formalin tissue", the "in paraffin tissue" and "dewaxed tissue" preparations are shown in blue, red, grey and green, respectively.

As a result of the discriminant analysis calculation, confusion matrices are generated, which summarize the correctly and falsely assigned FTIR spectra (predicted spectra) and correlate them to their known affiliation (actual spectra). The total number of FTIR spectra included in the PCA-DA model for each preservation procedure is illustrated in the matrix columns (actual), whereas the assigned FTIR spectra of each method are listed in each matrix row (predicted). Using this terminology, false positive/negative predictions and true positive/negative assignments of the FTIR spectra can be identified. An overview of the obtained confusion matrix for each parotid tissue type is presented in Table S1 – S3. For the PCA-DA models, where “native” and “formalin” are combined as one group the confusion matrixes are listed in Table S4 – S6.

Table S1 Confusion matrix salivary gland tissue PCA-DA with four classes.

**Confusion matrix salivary gland tissue PCA-DA**		**Actual**			
		in paraffin	formalin	native	dewaxed
**Predicted**	in paraffin	270	0	0	0
	formalin	0	192	96	9
	native	0	77	174	0
	dewaxed	0	1	0	260

Table S2 Confusion matrix of the Warthin tumor PCA-DA with four classes.

**Confusion matrix Warthin tumor PCA-DA**		**Actual**			
		in paraffin	formalin	native	dewaxed
**Predicted**	in paraffin	150	0	0	0
	formalin	0	131	47	0
	native	0	19	103	0
	dewaxed	0	0	0	150

Table S3 Confusion matrix pleomorphic adenoma PCA-DA with four classes.

**Confusion matrix pleomorphic adenoma PCA-DA**		**Actual**			
		in paraffin	formalin	native	dewaxed
**Predicted**	in paraffin	120	4	2	0
	formalin	0	84	69	1
	native	0	31	48	5
	dewaxed	0	1	1	114

Table S4 Confusion matrix salivary gland tissue PCA-DA with three classes.

**Confusion matrix salivary gland tissue PCA-DA**		**Actual**		
		in paraffin	formalin + native	dewaxed
**Predicted**	in paraffin	270	0	0
	formalin + native	0	540	15
	dewaxed	0	0	254

Table S5 Confusion matrix of the Warthin tumor PCA-DA with three classes.

**Confusion matrix Warthin tumor PCA-DA**		**Actual**		
		in paraffin	formalin + native	dewaxed
**Predicted**	in paraffin	150	1	0
	formalin + native	0	299	0
	dewaxed	0	0	150

Table S6 Confusion matrix pleomorphic adenoma PCA-DA with three classes.

**Confusion matrix pleomorphic adenoma PCA-DA**		**Actual**		
		in paraffin	formalin + native	dewaxed
**Predicted**	in paraffin	120	0	0
	formalin + native	0	231	6
	dewaxed	0	2	114

Table S7. Model quality parameters for the salivary gland tissue, Warthin tumor and pleomorphic adenoma PCA-DA models. "Native" and "formalin" are combined as one class. The robustness of the models can be deduced from the total and percentage amount of correctly assigned FTIR spectra for each preservation method. Based on this assignment, the average model parameters accuracy, sensitivity, specificity and precision can be calculated.

Entity	Fixation method	Total spectra	Correctly predicted	Correctly predicted / %	Accuracy / %	Sensitivity / %	Specificity / %	Precision / %
salivary gland tissue	in paraffin	270	270	100	99	98	99	99
	formalin + native	540	540	100				
	dewaxed	269	254	94				
Warthin tumor	in paraffin	150	150	100	100	100	100	100
	formalin + native	300	299	100				
	dewaxed	150	150	100				
pleomorphic adenoma	in paraffin	120	120	100	97	97	98	97
	formalin + native	233	231	99				
	dewaxed	120	114	95				
